# Monoterpenoids from *Artemisia austriaca* essential oil disrupt hormone‐mediated reserve mobilization to suppress *Avena fatua* seed germination

**DOI:** 10.1002/ps.70043

**Published:** 2025-07-15

**Authors:** Mohammad Pouresmaeil, Ali Movafeghi, Mohsen Sabzi‐Nojadeh, Morteza Kosari‐Nasab, Filippo Maggi

**Affiliations:** ^1^ Department of Plant, Cell and Molecular Biology, Faculty of Natural Sciences University of Tabriz Tabriz Iran; ^2^ Ahar Faculty of Agriculture and Natural Resources University of Tabriz Ahar Iran; ^3^ Drug Applied Research Center Tabriz University of Medical Sciences Tabriz Iran; ^4^ Chemistry Interdisciplinary Project (ChIP) Research Center, School of Pharmacy University of Camerino Camerino Italy

**Keywords:** germination, monoterpenoids, biocontrol, lipids, sugars, starch, weed

## Abstract

**BACKGROUND:**

The search for safe and effective alternatives to synthetic herbicides is crucial for sustainable agriculture. Essential oils and their bioactive monoterpenoids are promising candidates owing to their potent phytotoxic properties. This study aimed to characterize the essential oil of *Artemisia austriaca*, investigate the absorption of its main components, camphor and 1,8‐cineole, into *Avena fatua* (wild oat) seeds, and elucidate the mechanisms responsible for germination inhibition.

**RESULTS:**

The essential oil of *A. austriaca* was dominated by camphor (26.22%) and 1,8‐cineole (20.15%). These monoterpenoids were absorbed by *A. fatua* seeds, inhibiting germination in a concentration‐dependent manner. Their mode‐of‐action involved disruption of hormonal balance, significantly decreasing the gibberellic acid level (by ≤46.22% at 150 μg mL^−1^) while increasing the abscisic acid content (≤22.25‐fold at 150 μg mL^−1^). The compounds also inhibited key metabolic enzymes, *α*‐amylase and lipase, by ≤97.51% and 22.25% at 150 μg mL^−1^, respectively. This enzymatic inhibition led to the accumulation of energy reserves such as starch (≤9.92‐fold at 150 μg mL^−1^) and a corresponding decrease in available free sugars (by ≤84.56% at 150 μg mL^−1^).

**CONCLUSION:**

Camphor and 1,8‐cineole inhibit germination by disrupting hormonal signaling and blocking the metabolic pathways required for the mobilization of reserves in seeds. These findings clarify the specific phytotoxic mechanisms of these compounds, highlighting their potential as effective bioherbicides. This research advances the development of natural, eco‐friendly alternatives for sustainable weed management. © 2025 The Author(s). *Pest Management Science* published by John Wiley & Sons Ltd on behalf of Society of Chemical Industry.

## Graphical abstract

Terpenoid compounds from the essential oil of *Artemisia austriaca*, particularly camphor and 1,8‐cineole, infiltrate seed tissues and impede the germination of *Avena fatua* (wild oat) seeds by disrupting gibberellic acid and abscisic acid levels, as well as inhibiting critical enzymes involved in starch, lipid and protein metabolism.

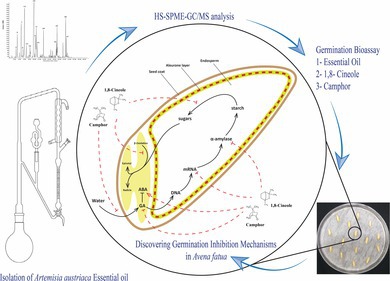

## 1 INTRODUCTION

Weed management continues to be one of the most significant challenges in global agriculture. Synthetic herbicides mainly serve as the predominant strategy for the control of invasive plants and the protection of crops. Although synthetic herbicides provide quick and cost‐effective solutions, they are facing growing criticism owing to environmental concerns, the development of herbicide‐resistant weeds, and potential risks to soil safety and non‐target species health.^1^, ^2^ Recently, bioherbicides originating from natural sources, particularly from plants, are gaining recognition as sustainable alternatives. These eco‐friendly agents inhibit the growth and development of weeds by impairing key biochemical and physiological processes, especially by inducing oxidative stress.^3^, ^4^, ^5^

Essential oils (EOs), primarily composed of volatile terpenoids, are attracting growing attention for their potential use as bioherbicides. These compounds demonstrate multiple mechanisms of action, thereby decreasing the probability of weed resistance. Moreover, they are biodegradable and possess low toxicity to mammals.^6^, ^7^ Recently, EOs derived from species belonging to the genus *Artemisia* L. (Asteraceae), including *Artemisia austriaca* Jacq.,^5^ *Artemisia absinthium* L.,^8^ *Artemisia fragrans* Willd.,^9^ *Artemisia herba‐alba* Asso,^10^ *Artemisia sieversiana* Ehrh. ex Willd.^11^ and *Artemisia vulgaris* L.,^12^ have been recognized for their potent weed‐killing properties, presenting prospects for the development of bioherbicides.

Germination is a crucial process in the life cycle of plants, involving the activation of metabolic pathways essential for the growth of embryos and the subsequent emergence of seedlings.^13^ Although extensive research has shed light on the physiological and biochemical mechanisms associated with later developmental stages, such as seedling growth and the maturation process, the germination phase has not been thoroughly investigated as a potential target for bioherbicide action. During germination, seeds undergo various metabolic procedures, including the hydrolysis of stored macromolecules like proteins, lipids, and polysaccharides. Additionally, changes in hormonal balance occur, which support cellular respiration and embryonic growth, as the germination stage lacks photosynthesis.^14^ After the process of imbibition, gibberellic acid (GA) is released from the aleurone layer, inducing the transcription of amylase genes. This leads to the hydrolysis of starch into simple sugars. Furthermore, peptidases and lipases become activated, facilitating the breakdown of proteins and lipids, respectively. At the same time, the *β*‐oxidation of fatty acids is initiated to ensure an adequate supply of sugars for the embryo.^15^

*Artemisia austriaca* Jacq., a perennial species distributed across Central Asia, Iran, China and Turkey, has demonstrated the presence of EOs with major bioactive compounds. Phytochemical characterization of its EOs by GC‐MS analysis led to identifying camphor (21.62%) and 1,8‐cineole (13.45%) as the primary constituents.

Previous studies have recognized various mechanisms by which terpenoids suppress seedling development. However, many aspects of the postimbibition germination processes at the seed level remain unclear and inadequately investigated. Although Pouresmaeil *et al*.^5^ documented postgerminative oxidative damage in *A. fatua* seedlings, the present study focuses exclusively on pre‐radicle emergence events at the seed level, particularly hormonal regulation and reserve mobilization.

Our previous work^5^ has shown that camphor and 1,8‐cineole induce oxidative stress in emerged *A. fatua* seedlings. However, when macromolecular reserves (starch, lipids, proteins) and hormones [GA, abscisic acid (ABA)] critically determine radicle emergence, the seed germination stage remains poorly understood as a target for monoterpenoid action. Whereas our prior study demonstrated that camphor and 1,8‐cineole induce reactive oxygen species (ROS) accumulation and lipid peroxidation in emerged *A. fatua* seedlings, the present work hypothesized that infiltration of these monoterpenoids into imbibed seeds disrupts the GA/ABA balance, inhibits α‐amylase and lipase, and halts reserve mobilization. All of these actions effectively inhibit the germination progression at the seed level. Thus, this study intends to systematically investigate (1) the penetration efficiency of EO of *A. austriaca* (EOAA) and its major volatiles, camphor and 1,8‐cineole, into the seed tissues of *A. fatua* using headspace solid‐phase microextraction coupled with gas chromatography–mass spectrometry (HS‐SPME‐GC‐MS); and (2) the biochemical and physiological mechanisms underlying the inhibition of seed germination of *A. fatua* by EOAA. We propose that the monoterpene‐rich composition of EOAA facilitates its rapid absorption through the seed coat, resulting in an imbalance between GA and ABA levels. Such a disruption may inhibit the activity of *α*‐amylase, promote the accumulation of starch, reduce sugar levels, and change the activity of lipase as well as the composition of fatty acids.

## 2 MATERIALS AND METHODS

### 2.1 Preparation of plant samples

*Artemisia austriaca* was collected from Varzaqan County in East Azerbaijan Province, Iran, to isolate its EO in the summer of 2022. The seeds of *A. fatua* also were gathered from the same location and used as a model weed to assess the phytotoxic activity of the acquired EO and its major volatile compounds.

### 2.2 Essential oil isolation and analysis by HS‐SPME‐GC/MS

The EOAA was obtained from the flowering aerial parts using a Clevenger‐type apparatus. Fresh plant material, weighing 150 g, was finely chopped into pieces measuring <1 cm and subjected to hydro‐distillation for 4 h using 2 L of distilled water. This procedure was repeated for three separate plant samples to ensure consistency. The obtained EOAA was collected in a dark glass vial and stored at 4 °C until further bioassay analyses.

For chemical profiling, 10 μL EOAA was included in a headspace vial and sealed with a septum to prevent evaporation. The sample was heated at 40 °C for 10 min, and subsequently a polydimethylsiloxane (PDMS) SPME fiber (needle size = 24 gauge, df = 100 μm) (Supelco/Merck, Darmstadt, Germany) was inserted into the headspace of the vial using a manual holder (Supelco). The fiber was exposed to the headspace for 20 min to promote the absorption of volatile compounds. After absorption, the SPME fiber was manually injected into the injection port of a gas chromatography–mass spectrometry (GC‐MS) system for desorption of the volatiles. Desorption was performed at 250 °C for 5 min in splitless mode, following the manufacturer's recommended procedure.

GC‐MS analysis was performed using an 7890B gas chromatograph coupled to a 5977A mass selective detector (both from Agilent Technologies, Santa Clara, CA, USA). The separation was conducted on an HP‐5MS capillary column (30 m × 0.25 mm inner diameter, 0.25 μm film thickness; Agilent Technologies), with helium used as the carrier gas at a flow rate of 1 mL min^−1^. The oven temperature was programmed as follows: an initial temperature of 40 °C was maintained for 4 min, then increased to 160 °C at a rate of 5 °C min^−1^, followed by a further increase to 200 °C at a rate of 20 °C min^−1^, and held at 200 °C for 2 min. The injector temperature was set to 250 °C, and the mass spectrometer was operated with a transfer line temperature of 280 °C. The mass spectrometer functioned in electron ionization mode at 70 eV, with a mass scan range of 5–500 *m/z*. Compound identification was achieved by comparing the obtained mass spectra with reference spectra in the NIST05 and Wiley7 mass spectral libraries and by calculating retention indices using *n*‐alkanes analyzed under the same conditions. Data analysis, encompassing the calculation of peak areas and comparison of mass spectra, was performed using Agilent chemstation software. The analysis of chemical composition was performed for three distinct samples and reported as mean ± SD.

### 2.3 Experimental design and treatments

The treatments consisted of various concentrations (0, 25, 50, 100, 150 and 200 μg mL^−1^) of the EOAA, 1,8‐cineole (Fluka, Seelze, Germany), and camphor (Merck). All experiments were conducted using a completely randomized design (CRD), with three replicates for each treatment. The desired concentrations were achieved by dissolving the compounds in distilled water, with a trace amount of Tween 80® (Merck) added as a surfactant to ensure proper dispersion. Distilled water served as the control treatment.

### 2.4 Bioassay

Uniform seeds of *A. fatua* were carefully selected and subjected to surface sterilization by immersing them in a 5% sodium hypochlorite solution for 5 min. This process was followed by extensive rinsing with distilled water to eliminate any remaining sterilizing agent. Seeds exhibiting high germination potential were identified by performing a flotation test in water. During preparation of the bioassay, a filter paper layer was positioned within Petri dishes with a diameter of 100 mm, followed by adding 10 mL of each concentration. The seeds were then arranged on moistened filter paper, and the Petri dishes were sealed with parafilm to minimize evaporation and preserve humidity levels. The Petri dishes were kept in complete darkness for 48 h to promote germination. Subsequently, the seeds were transferred to a controlled environment with a 16 h:8 h, light:dark photoperiod for 10 days. At the end of this period, the seedlings were harvested.

### 2.5 Germination‐related parameters

Germination‐related parameters, such as germination index and germination speed index, were calculated using the following formula, as described by Li *et al*.^16^ The germination percentage of the *A. fatua* exposed to EOAA, 1,8‐cineole, and camphor was reported previously.^5^

$$\text{Germination index}=\sum \frac{G_{t}}{D_{t}}$$

where *G*_*t*_ is the germinated seeds on day *t*, and *D*_*t*_ is the day on which the germinated seeds are counted.

$$\text{Germination speed index}=\sum \left(n\times X_{n}\right)$$

where *n* is the day on which the germinated seeds are counted, and *X*_*n*_ is the number of germinated seeds on day *n*.

### 2.6 HS‐SPME‐GC‐MS analysis of the absorbed compounds by seeds

The HS‐SPME‐GC‐MS technique was utilized to investigate the absorption of volatile compounds into the seeds of *A. fatua*. Briefly, the volatile‐treated seeds were surface‐washed with 2 mL methanol (Merck) and *n*‐hexane (Merck) to remove any external contaminants (washing was repeated for three times for each solvent). Thereafter, the seeds were surface‐dried under a laminar flow hood. They were then homogenized with 200 μL phosphate buffer and transferred into a headspace vial. The vial was immediately sealed with a septum to prevent the loss of volatile compounds through evaporation. The HS‐SPME procedure was the same as the EOAA analysis, but the oven temperature condition was quite different as a consequence of minimizing the analysis time and focusing on the major compounds such as 1,8‐cineole and camphor. The oven temperature program began at 70 °C, held for 3 min, then increased to 140 °C at a rate of 10 °C min^−1^, and finally held at 140 °C for an additional 2 min, resulting in a total run time of 12 min. The GC‐MS system and parameters were the same as those described in Section 2.2.

### 2.7 Quantification of total soluble protein content

Total soluble protein content was quantified using the Bradford method with slight modifications.^17^ Briefly, the seeds were homogenized in 50 mm phosphate buffer (pH = 7) and centrifuged at 10000 × *g* for 5 min. An amount of 100 μL supernatant was pooled with 1000 μL Bradford reagent and kept at room temperature (RT) for 60 min. The absorbance of the samples was measured at 595 nm, and the content of total soluble proteins was expressed as mg g^−1^ based on the standard curve prepared with bovine serum albumin (BSA).

### 2.8 Quantification of gibberellic acid and abscisic acid

For the extraction of GA and ABA, the plumule and radicle of the seedling were excised, and the remaining seed material was thoroughly homogenized in ice‐cold phosphate‐buffered saline (100 mm, pH 7). The homogenate was then centrifuged at 10000 × *g* for 5 min to separate the supernatant from the solid debris. The resulting supernatant was carefully collected and filtered through a 0.22‐μm syringe filter to remove any particulate matter.^18^ The concentrations of GA and ABA in the filtered supernatant were quantified using an enzyme‐linked immunosorbent assay (ELISA) kit (YuDuo Biotechnology Co., Ltd, Shanghai, China), following the manufacturer's instructions.

### 2.9 Quantification of total starch content and α‐amylase activity

The starch content was assessed using the starch‐iodine method.^19^ The seeds were homogenized in 1 mL distilled water using a homogenizer. The resulting homogenate was boiled for 10 min and then immediately cooled to RT. The solution was centrifuged at 10000 × *g*, and 300 μL of supernatant were transferred to a new tube containing 700 μL ethanol (100%). The mixture underwent a second centrifugation to precipitate the starch. For color development, 50 μL of a KI:I_2_ solution (20 g L^−1^ potassium iodide and 2 g L^−1^ iodine) (both from Merck) were added to 950 μL supernatant. The mixture was subjected to a 30‐min incubation period to ensure the stabilization of the color change. The absorption of samples was measured at 594 nm using a spectrophotometer. Pure starch was used as a standard for calibration, with concentrations ranging from 0 to 2.5 mg mL^−1^, to quantify the starch content in the samples.

In order to quantify *α*‐amylase activity, the starch‐iodine method was applied, as detailed by Xiao *et al*.,^20^ with slight modifications. *α*‐Amylase was extracted using a 50 mm phosphate buffer (pH 7) and centrifuged at 10000 × *g* for 5 min. The resulting supernatant was used as the enzyme source. The reaction mixture consisted of 200 μL enzyme extract, 200 μL phosphate buffer (100 mm, pH 7), and 200 μL starch (Soluble starch; Sigma Aldrich, Taufkirchen, Germany) solution (2 g L^−1^). The mixture was incubated at 50 °C for 30 min to allow the enzymatic reaction to proceed. Afterwards, 50 μL of 1 m HCl (Merck) were added to terminate the reaction. Subsequently, 350 μL of a reagent containing I_2_ (5 mm) + KI (5 mm) were added to the mixture, which was then allowed to stand for 15 min to ensure complete color development. The absorbance of the samples was measured at 580 nm using a spectrophotometer. The activity of *α*‐amylase was calculated according to the following formula:

$$\begin{array}{l} \text{Activity}\left(\mathrm{\mu g}\;\text{starch}\;{\mathrm{mg}}^{-1}\;\text{protein}\;\min\;{\mathrm{mg}}^{-1}\right)\\ \;=\frac{\left(A_{580}\;\text{control}–A_{580}\;\text{sample}\right)\times \mathrm{RV}}{A_{580}\;\text{of}\;1\frac{\mathrm{mg}}{\mathrm{mL}}\;\text{starch}\times \Delta t\times \mathrm{VE}\times d}\left(\frac{1}{\mathrm{mg}\;\text{protein}}\right) \end{array}$$

where *A*_580_ is the absorbance of the samples at optical density (OD) = 580 nm. RV is reaction volume. *A*_580_ of 1 mg mL^−1^ starch is the absorbance of the 1 mg mL^−1^ according to the standard curve prepared with different concentrations of starch. Δ_*t*_ is the time of reaction, VE is the volume of enzyme extract and *d* is the cuvette volume. Concentration of protein is given in mg g^−1^ fresh weight (FW).

### 2.10 Assessment of total soluble, reducing and nonreducing sugar content

The total soluble sugar content was determined using the phenol‐sulfuric acid method.^21^ Total soluble sugars were extracted using ethanol (80%) (Merck) and centrifuged at 10000 × *g* for 15 min. An amount of 250 μL supernatant was mixed with 250 μL phenol (5%) (Merck), and immediately 500 μL concentrated sulfuric acid (Merck) were added. After 10 min, the content in the tubes was placed in a water bath at 25 °C for 20 min and then the absorbance of the samples was recorded at 490 nm. The content of total soluble sugars was expressed as mg g^−1^ FW using the standard curve of d‐glucose (Analytical standard; Sigma Aldrich).

Reducing sugars were extracted using 70% ethanol with a homogenizer. The resulting homogenate was centrifuged at 10000 × *g*, and the supernatant was collected as the source of reducing sugars. The reducing sugar assessment was performed following the procedure established by Krivorotova & Sereikaite,^22^ with slight modifications, utilizing the 3,5‐dinitrosalicylic acid (DNS) reagent (Sigma Aldrich). The DNS reagent (1%) was freshly prepared by dissolving 1 g DNS in 80 mL sodium hydroxide (0.5 M) (Merck) containing 30 g sodium‐potassium tartaric acid (Merck) at 45 °C. The solution was subjected to heating and stirring until it was fully dissolved. Subsequently, it was allowed to cool to RT and then adjusted to a final volume of 100 mL with distilled water. For color development, 800 μL DNS reagent was mixed with 200 μL supernatant in a test tube. The mixture was heated at 95 °C for 5 min to allow the reaction to proceed, followed by cooling to RT. The absorbance of the samples was then measured at 540 nm using a spectrophotometer. The concentration of reducing sugars was determined based on a standard curve prepared using d‐glucose solutions with concentrations ranging from 0 to 100 μg mL^−1^. The reducing sugar content was expressed as mg g^−1^ FW.

Non‐reducing sugar content was calculated according to the following formula:

$$\begin{array}{l} \text{Non‐rducing sugar content}\left(\mathrm{mg}\;g^{-1}\;\mathrm{FW}\right)\\ \;=\text{total soluble sugars}–\text{reducing sugars.} \end{array}$$

### 2.11 Quantification of total lipid content and lipase activity

The sulfo‐phospho‐vanillin method was employed to quantify the total lipid content in the seed samples.^23^ The phospho‐vanillin reagent was prepared by dissolving 120 mg vanillin (Sigma Aldrich) in 20 mL distilled water, followed by adjusting the final volume to 100 mL with phosphoric acid (Merck). The extraction procedure for total lipids was consistent with the described method in Section 2.11. Briefly, 40 μL supernatant obtained from the extraction process were dried in a microtube at 40 °C. Subsequently, 200 μL sulfuric acid were added to the dried sample, and the mixture was heated at 100 °C for 10 min. Following the cooling process, 800 μL of the phospho‐vanillin reagent were introduced into the solution, which was then incubated at 37 °C for 15 min to allow the reaction to proceed. The absorbance of the samples was measured at 530 nm using a spectrophotometer. A standard curve was prepared using oleic acid (Sigma Aldrich) solutions with concentrations ranging from 0 to 120 μg mL^−1^ to quantify the total lipid content. The total lipid content in the seed samples was ultimately expressed as mg g^−1^ FW.

Lipase activity was assessed using Tween 80® as the substrate. The extraction of lipase was conducted by homogenizing the sample in a phosphate buffer solution (50 mm, pH 7). This was followed by centrifugation at 10000 × *g* to isolate the supernatant, which served as the source of the enzyme. The reaction mixture consisted of 1000 μL Tween 80 (1% in 20 mm Tris–HCl) (Tris form; Merck), 900 μL CaCl_2_ (80 mm) (Merck), and 100 μL enzyme extract (supernatant). The reaction was initiated by mixing these components, and the increase in absorbance—corresponding to the production of calcium oleate—was monitored at 450 nm over 3 min. Lipase activity was quantified based on the change in absorbance, using an extinction coefficient of 1975 m ^−1^ cm^−1^ for calcium oleate. The activity was expressed as μmol oleic acid mg^−1^ protein min^−1^ ^24^ calculated according to the following formula:

$$\begin{array}{l} \text{Activity}\left(\mathrm{\mu g}\;\text{starch}\;{\mathrm{mg}}^{-1}\;\text{protein}\;\min^{-1}\right)\\ \;=\left(\frac{\left(\Delta \;A_{450}\right)\times \mathrm{RV}}{\Delta t\times \mathrm{EC}\times \mathrm{VE}\times d}\right)\times \left(\frac{1}{\mathrm{mg}\;\text{protein}}\right) \end{array}$$

where Δ *A*_450_ is the change in absorbance of the samples at OD = 450 nm. RV is the reaction volume, Δ_*t*_ is the time of reaction, EC is the extinction coefficient, VE is the volume of enzyme extract and *d* is the cuvette volume. Concentration of protein is given in mg g^−1^ FW.

### 2.12 Seed fatty acid composition

Fatty acids were extracted from seed samples following the method described by Folch *et al*.,^25^ with slight modifications. The seeds were homogenized in 2 mL chloroform:methanol solution (2:1 ratio) (Merck) using a homogenizer operating at 7500 rpm. The resulting mixture was centrifuged at 10000 × *g* for 5 min, and the supernatant was carefully collected. To separate the lipid phase, 2 mL of a 0.73% sodium chloride (NaCl) (Merck) solution were added to 1 mL supernatant, and the mixture was thoroughly shaken. Afterwards, the chloroform layer, containing the lipids, was retrieved. Subsequently, 1 mL of the chloroform layer was mixed with 2 mL of 1% sulfuric acid in methanol. This solution was heated at 50 °C for 12 h to promote the conversion of fatty acids into their corresponding methyl esters.^26^ Following the incubation period, the solution was re‐extracted using 2 mL of 0.73% NaCl solution, and the chloroform phase was collected again. The resulting solution was evaporated to dryness at 40 °C, and 200 μL *n*‐hexane was added to dissolve the dried residue.

The fatty acid composition of *A. fatua* seed samples was analyzed using the same GC‐MS instrument and HP‐5MS column described in Section 2.2. A volume of 1 μL of the prepared sample was injected into the GC‐MS system. Helium was used as the carrier gas at a flow rate of 1 mL min^−1^. The oven temperature program was as follows: the initial temperature was set at 60 °C and held for 30 min, then increased to 220 °C at a rate of 5 °C min^−1^, and finally held at 220 °C for an additional 10 min. After analyzing the fatty acid composition, the concentration of each fatty acid was calculated based on its density and expressed as mg g^−1^ FW.

### 2.13 Statistical analysis

The data were analyzed using Spss v22 software. Mean comparisons were performed using the Duncan multiple range test within the general linear model (GLM) at a significance level of *P ≤* 0.05. Before the mean comparison, all acquired data were subjected to a normality test. All experiments were conducted as CRD with three replications and shown as mean ± standard deviation. Graphical representations of the data were prepared using excel 2019 (Microsoft, Redmond, WA, USA).

## 3 RESULTS

### 3.1 Analysis of EOAA by HS‐SPME‐GC/MS

**Figure 1:**
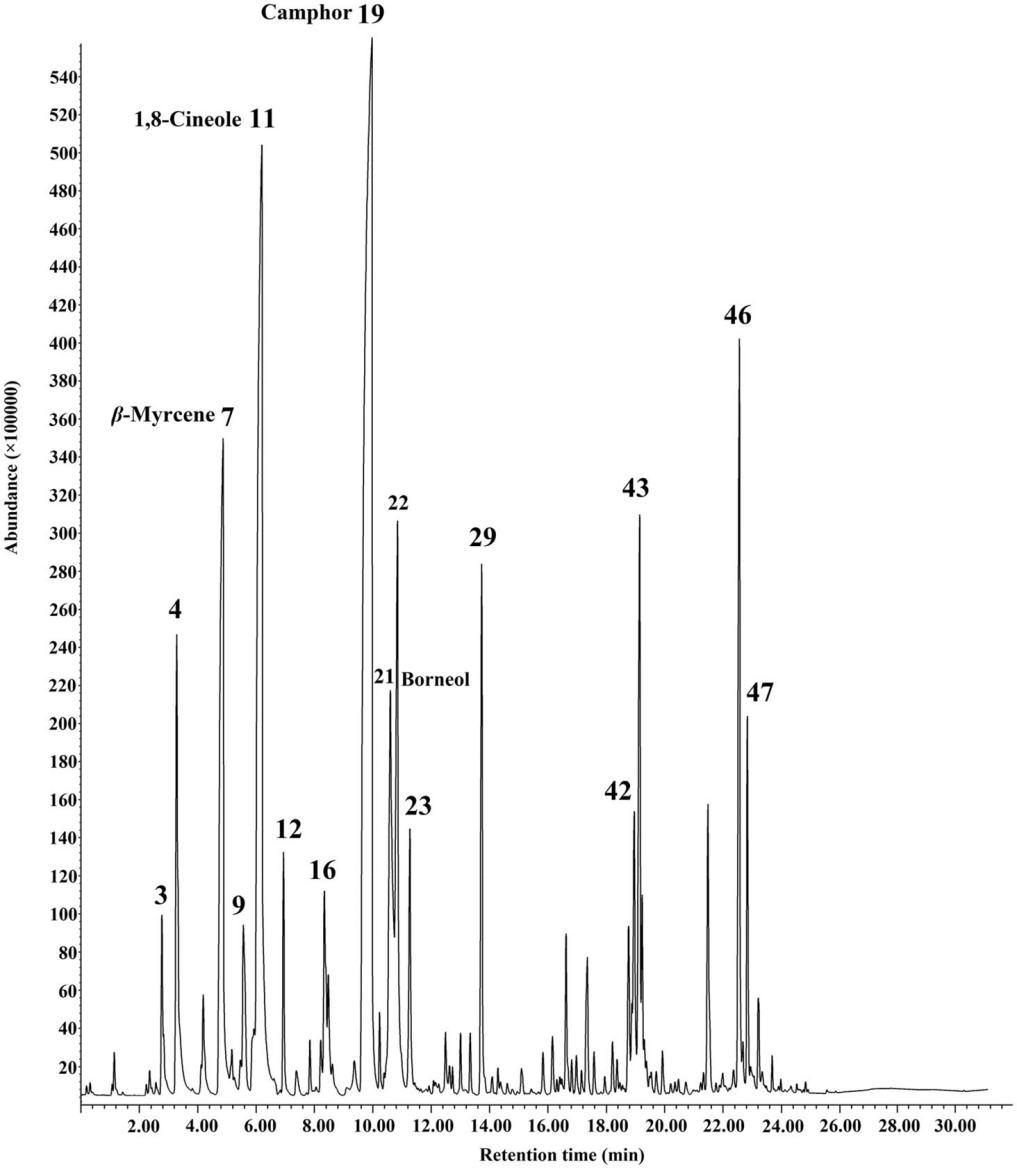
Total ion chromatogram (TIC) of essential oil of *Artemisia austriaca* obtained by HS‐SPME‐GC‐MS and PDMS fiber.

The HS‐SPME‐GC‐MS analysis of the EOAA recognized a number of compounds (Fig. 1), of which 48 were identified (Table 1). The main compounds identified were camphor (26.22%), 1,8‐cineole (20.15%), *β*‐myrcene (8.32%) and borneol (6.26%). The EOAA was primarily composed of oxygenated monoterpenes (64.97%). However, it also contained other classes of compounds, including monoterpene hydrocarbons (17.30%), sesquiterpene hydrocarbons (11.77%) and oxygenated sesquiterpenes (2.22%).

**Table 1:** The chemical composition of essential oil of *Artemisia austriaca* analyzed by HS‐SPME‐GC‐MS

No.	Compounds	RI^a^_Rep_	RI^b^_Cal_	Peak area^**c**^ (%)
1	1‐Butanol, 2‐methyl‐, acetate	888	889	0.15 ± 0.01
2	Tricyclene	926	926	0.13 ± 0.04
3	*α*‐Pinene	**940**	**942**	**1.03 ± 0.24** ^**d**^
4	Camphene	**952**	**950**	**3.31 ± 0.76**
5	Sabinene	973	973	0.11 ± 0.02
6	*β*‐Pinene	978	979	0.57 ± 0.27
7	*β*‐Myrcene	**981**	**980**	**8.32 ± 0.21**
8	*α*‐Phellandrene	996	995	0.08 ± 0.01
9	*α*‐Terpinene	**1017**	**1015**	**1.39 ± 0.13**
10	*p*‐Cymene	1021	1020	0.31 ± 0.43
11	1,8‐Cineole	**1046**	**1045**	**20.15 ± 7.09**
12	*γ*‐Terpinene	**1064**	**1067**	**1.75 ± 0.77**
13	*trans‐*Sabinene hydrate	1071	1069	0.29 ± 0.08
14	*α*‐Terpinolene	1085	1083	0.32 ± 0.08
15	Butanoic acid, 2‐methyl‐, 3‐methylbutyl ester	1102	1103	0.34 ± 0.05
16	*n*‐Amyl isovalerate	**1108**	**1107**	**1.49 ± 0.22**
17	*α*‐Campholenal	1128	1128	0.09 ± 0.01
18	2‐Cyclohexen‐1‐ol, 1‐methyl‐4‐(1‐methylethyl)‐, *trans*‐	1142	1143	0.14 ± 0.02
19	Camphor	**1147**	**1147**	**26.52 ± 0.88**
20	Pinocarvone	1165	1165	0.44 ± 0.16
21	Borneol	**1167**	**1166**	**6.26 ± 1.71**
22	Terpinen‐4‐ol	**1177**	**1178**	**4.18 ± 0.93**
23	*α*‐Terpineol	**1191**	**1191**	**2.34 ± 0.30**
24	Neral	1242	1141	0.38 ± 0.11
25	Carvotanacetone	1245	1245	0.19 ± 0.07
26	Isoamyl hexanoate	1250	1250	0.16 ± 0.08
27	Verbenyl acetate	1267	1265	0.36 ± 0.10
28	Geranial	1269	1268	0.40 ± 0.17
29	Bornyl acetate	**1280**	**1281**	**3.14 ± 0.16**
30	Undecanal	1305	1304	0.14 ± 0.04
31	Thymol	1306	1305	0.05 ± 0.00
32	Bicycloelemene	1324	1324	0.09 ± 0.02
33	Eugenol	1373	1372	0.25 ± 0.01
34	Copaene	1377	1378	0.34 ± 0.01
35	*β*‐Elemene	1394	1393	0.69 ± 0.23
36	*cis*‐Jasmone	1396	1396	0.13 ± 0.04
37	*trans*‐Isoeugenol	1449	1448	0.20 ± 0.04
38	Butyric acid, 2‐bornyl ester	–	1450	0.12 ± 0.01
39	*trans*‐Caryophyllene	1451	1452	0.90 ± 0.11
40	*α*‐Amorphene	1482	1481	0.24 ± 0.04
41	Aromadendrene	1485	1485	0.37 ± 0.04
42	Germacrene D	**1487**	**1487**	**1.48 ± 0.08**
43	*β*‐Selinene	**1488**	**1489**	**3.16 ± 0.25**
44	Bicyclogermacrene	1490	1491	0.05 ± 0.01
45	*β*‐Bisabolene	1505	1505	0.04 ± 0.01
46	*δ*‐Cadinene	**1515**	**1516**	**4.18 ± 0.41**
47	*β*‐Eudesmol	**1650**	**1649**	**1.65 ± 0.07**
48	*α*‐Bisabolol	1681	1680	0.25 ± 0.03
Total identified				**97.87 ± 1.51**
Monoterpene hydrocarbons				**17.30 ± 0.39**
Oxygenated monoterpenes				**64.97 ± 8.72**
Sesquiterpene hydrocarbons				**11.77 ± 1.06**
Oxygenated sesquiterpenes				**2.22 ± 0.42**
Others				**2.31 ± 0.13**

^a^ Reported retention indices.

^b^ Calculated retention indices based on the *n*‐alkanes' analysis.

^c^ Percentage of each peak area.

^d^ The bold values represent compounds with amounts of > 1% of the total composition.

### 3.2 Germination‐related parameters

**Figure 2:**
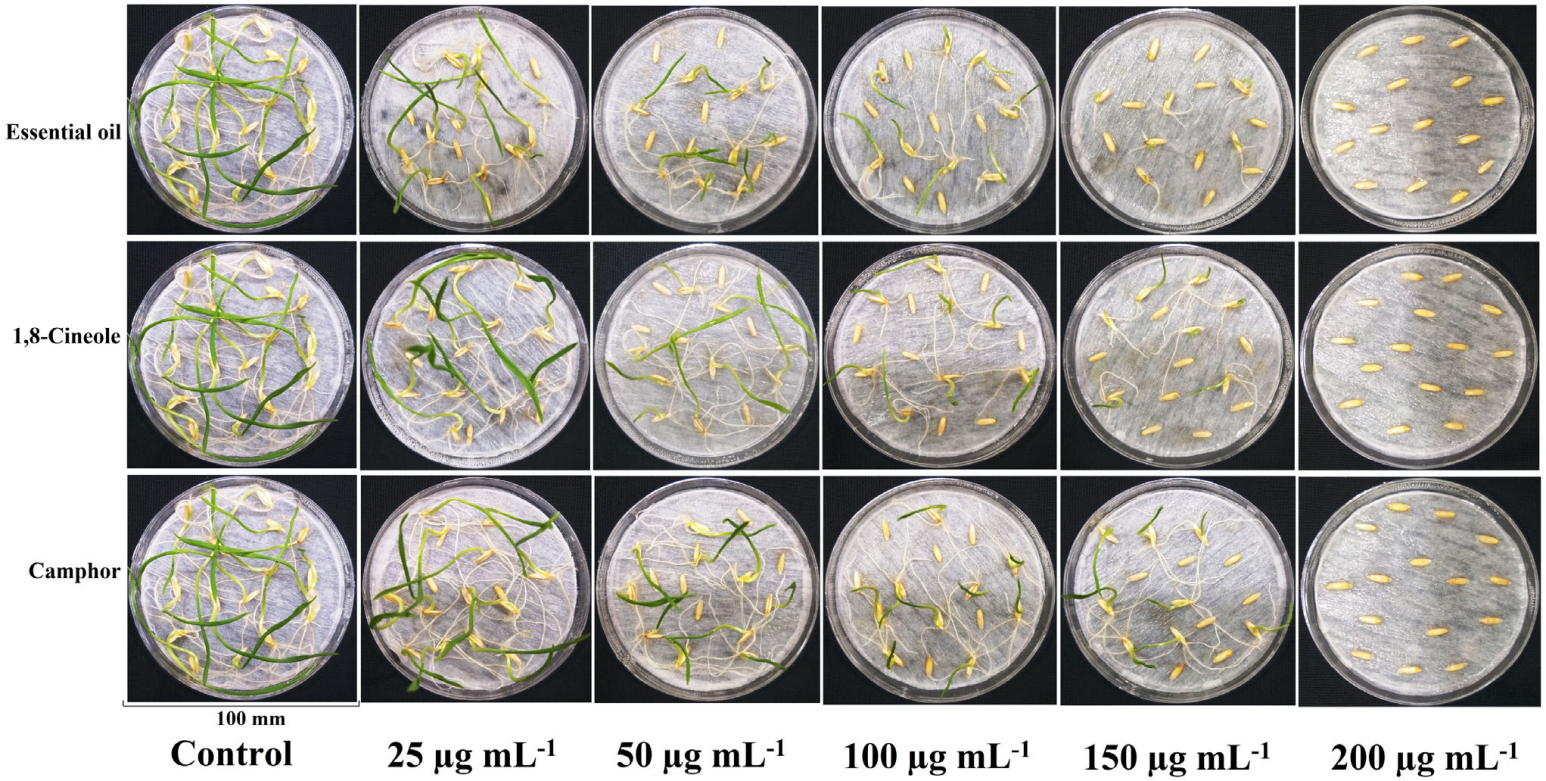
Germination of *Avena fatua* seeds exposed to different concentrations of *Artemisia austriaca* essential oil, camphor, and 1,8‐cineole after 7 days.

Figure 2 shows the effects of EOAA, 1,8‐cineole and camphor on the germination and seedling growth of *A. fatua*. It was observed that the germination percentage decreased in a concentration‐dependent manner and germination was fully suppressed at 200 μg mL^−1^ of EOAA, 1,8‐cineole and camphor.

Table 2 represents the germination index and germination speed index of *A. fatua* seeds exposed to EOAA, 1,8‐cineole and camphor. The germination index and germination speed index were reduced in a concentration‐dependent manner. At 200 μg mL^−1^ of all agents, the mentioned parameters were reduced by 100% compared to the control groups.

**Table 2:** Germination index and germination speed index of *Avena fatua* exposed to different concentrations of essential oil of *Artemisia austriaca*, 1,8‐cineole, and camphor

Treatments (μg mL^–1^)		Germination index	Germination speed index
Essential oil	Control	40.10 ± 0.58a	539.33 ± 0.58a
	25	25.93 ± 1.53b	377.67 ± 21.46b
	50	22.84 ± 0.75c	333.67 ± 9.24c
	100	18.94 ± 1.14d	279.67 ± 5.03d
	150	16.29 ± 1.57d	234.00 ± 20.78e
	200	00.000 ± 0.00e	00.00 ± 00.00f
1,8‐Cineole	Control	40.10 ± 0.58a	539.3 ± 30.58a
	25	35.19 ± 1.72b	482.67 ± 22.19b
	50	24.11 ± 2.90c	342.33 ± 25.50c
	100	21.34 ± 1.28c	294.67 ± 17.01d
	150	18.35 ± 1.36d	264.00 ± 19.29e
	200	00.00 ± 00.00e	00.00 ± 00.00f
Camphor	Control	40.10 ± 0.58a	539.33 ± 0.58a
	25	30.67 ± 3.30b	440.33 ± 44.06b
	50	22.78 ± 2.72c	328.67 ± 31.79c
	100	20.21 ± 2.56cd	292.67 ± 30.24d
	150	17.21 ± 2.25d	249.67 ± 24.58e
	200	00.00 ± 00.00e	00.00 ± 00.00f

Different letters in each column indicate statistically significant differences at *P* ≤ 0.05 as determined by Duncan's multiple range test. The data are presented as the mean of three replicates ± standard deviation.

### 3.3 Analysis of volatiles absorbed in seeds

**Figure 3:**
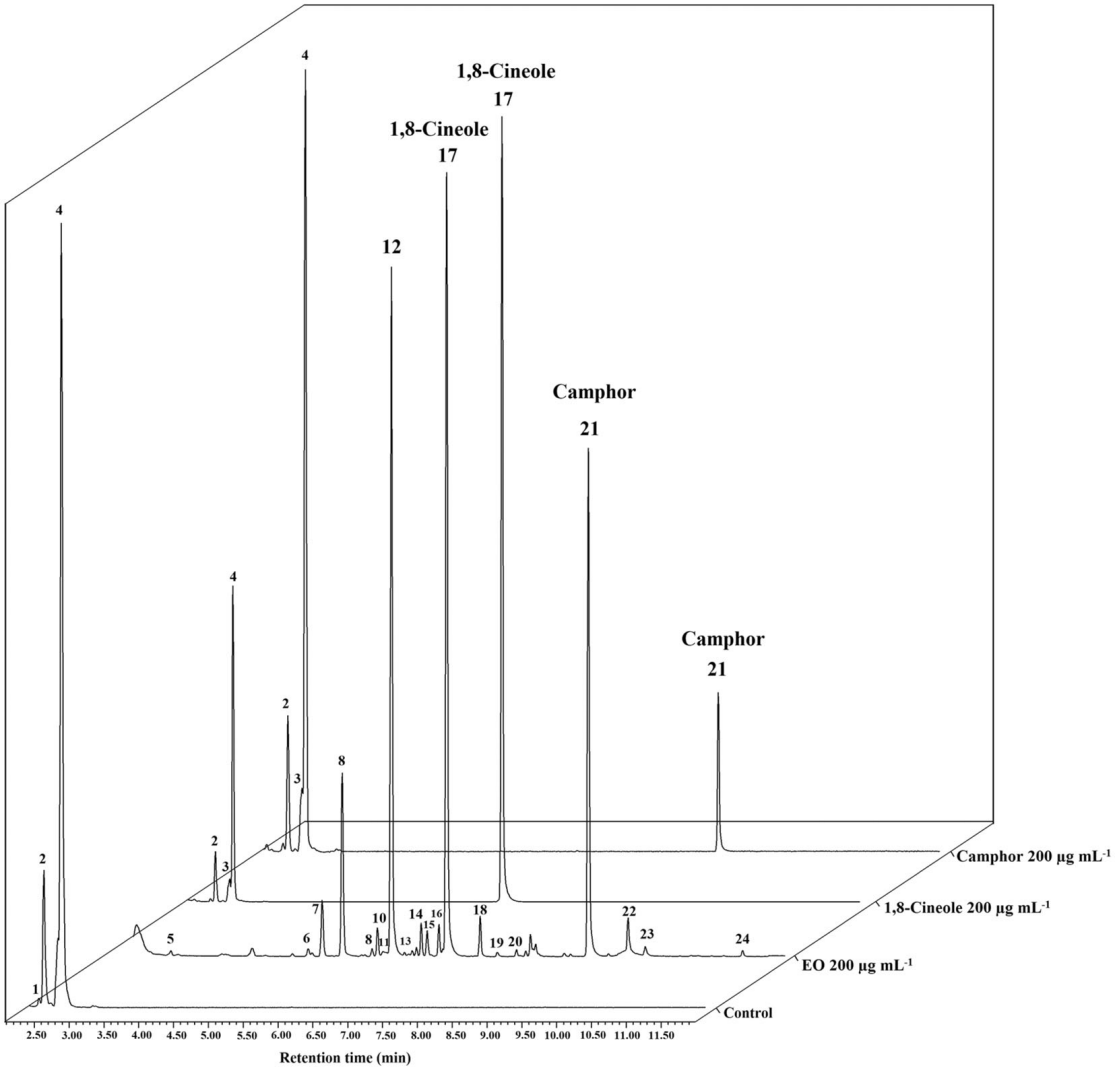
Total ion chromatograms (TIC) obtained by HS‐SPME‐GC‐MS for the identification of volatile constituents of *Artemisia austriaca* penetrating the *Avena fatua* seeds. The peaks were numbered according to the data presented in Table 3.

The HS‐SPME‐GC‐MS analysis of the *A. fatua* seed samples indicated that EOAA, including its primary constituents 1,8‐cineole and camphor, and 19 other compounds, can penetrate the seeds (Fig. 3; Table 3). As expected, seeds treated with 200 μg mL^−1^ of EOAA showed the presence of additional compounds originating from the EOAA, indicating the absorption of a broader range of compounds. Specifically, 1,8‐cineole and camphor were detected in seeds treated with 200 μg mL^−1^ of both compounds. By contrast, no volatile compounds related to the EOAA were identified in the control samples that had not undergone any treatment. This observation confirms that the detected compounds in the treated seeds originated exclusively from the applied treatments and not from any external contamination or background levels.

**Table 3:** Identified monoterpenoid compounds in the seeds of *Avena fatua* exposed to 200 μg mL^−1^ of essential oil of *Artemisia austriaca* (EOAA), 1,8‐cineole and camphor using HS‐SPME‐GC‐MS

No.	Compounds	Treatments			
		Control	EOAA (200 μg mL^−1^)	1,8‐cineole (200 μg mL^−1^)	Camphor (200 μg mL^−1^)
1	Heptane, 2‐methyl‐	+	−	−	+
2	Heptane, 3‐methyl‐	+	−	+	+
3	Cyclopentane, 1‐ethyl‐3‐methyl‐	−	−	+	+
4	Octane	+	−	+	+
5	Cyclooctane	−	+	−	−
6	Tricyclene	−	+	−	−
7	*α*‐Pinene	−	+	−	−
8	Camphene	−	+	−	−
9	Sabinene	−	+	−	−
10	*β*‐Pinene	−	+	−	−
11	1‐Octen‐3‐ol	−	+	−	−
12	*β*‐Myrcene	−	+	−	−
13	*α*‐Phellandrene	−	+	−	−
14	Butanoic acid, pentyl ester	−	+	−	−
15	*α*‐Terpinene	−	+	−	−
16	p‐Cymene	−	+	−	−
17	1,8‐Cineole	−	+	+	−
18	*γ*‐Terpinene	−	+	−	−
19	*trans*‐Sabinene hydrate	−	+	−	−
20	*α*‐Terpinolene	−	+	−	−
21	Camphor	−	+	−	+
22	Terpinen‐4‐ol	−	+	−	−
23	*α*‐Terpineol	−	+	−	−
24	Bornyl acetate	−	+	−	−

+ Detected, − Not detected.

### 3.4 Germination‐related phytohormones

**Figure 4:**
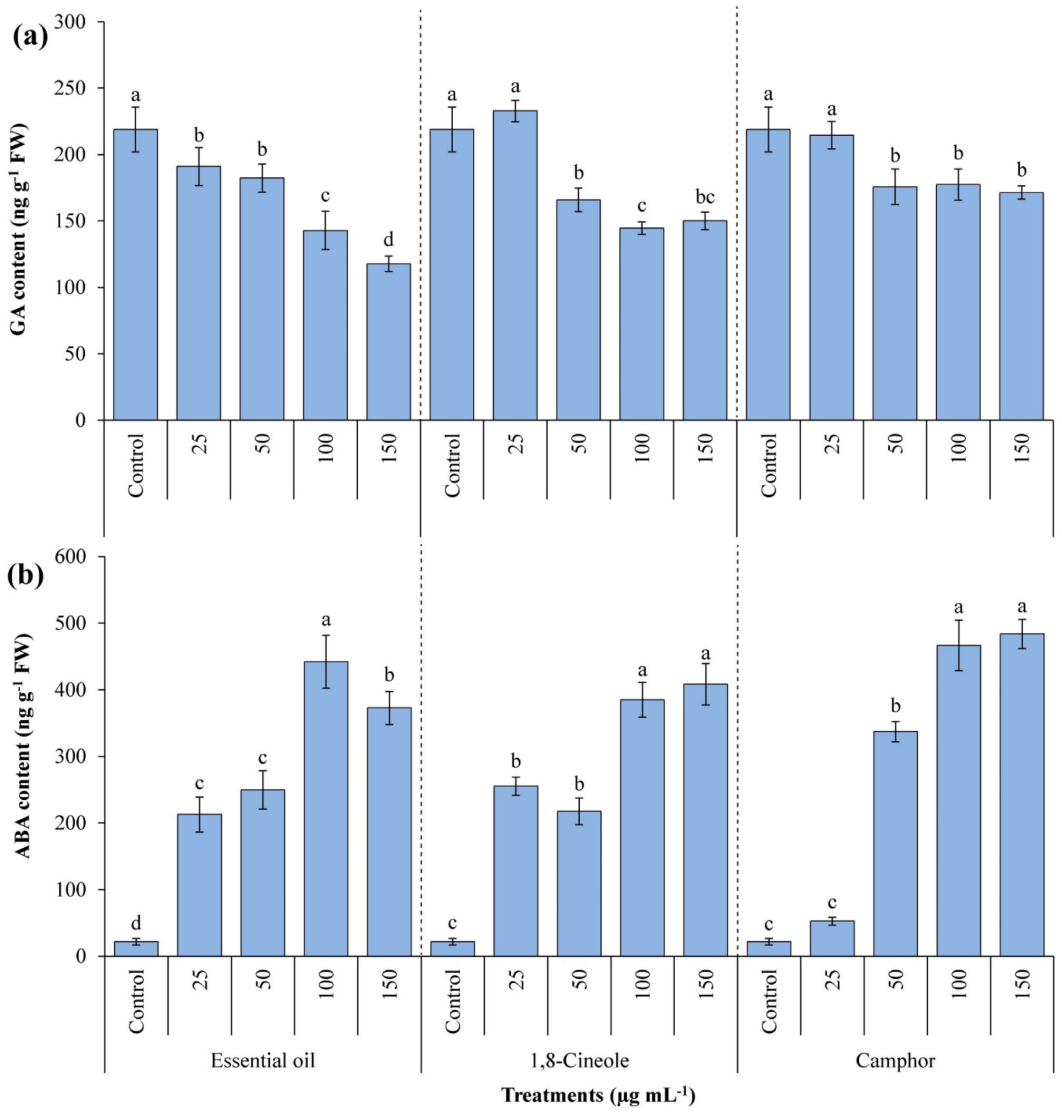
Content of gibberellic acid (a) and abscisic acid (b) in *Avena fatua* seeds after treatment with various concentrations of essential oil from *Artemisia austriaca*, 1,8‐cineole and camphor. Different letters on each column indicate statistically significant differences at *P ≤* 0.05 as determined by Duncan's multiple range test. The data are displayed as the mean of three replicates ± standard deviation.

Figure 4 presents the content of GA and ABA in *A. fatua* seeds treated with various concentrations of EOAA, 1,8‐cineole and camphor. The results demonstrate a significant reduction in GA content (*P ≤* 0.05) in seeds exposed to the volatile compounds at specific concentrations. Notably, seeds treated with 50, 100 and 150 μg mL^−1^ of the compounds exhibited markedly lower GA levels compared to the untreated control seeds. The most pronounced decrease in GA content was observed in seeds treated with 150 μg mL^−1^ of EOAA, 100 μg mL^−1^ of 1,8‐cineole and 150 μg mL^−1^ of camphor. These treatments resulted in reductions of 46.22%, 33.94% and 21.66%, respectively, relative to the control samples [Fig. 4(a)].

By contrast, the ABA content, which plays a key role in inhibiting germination, was significantly elevated (*P ≤* 0.05) in seeds treated with volatile compounds compared to the control. The highest ABA levels were observed in seeds treated with 100 μg mL^−1^ of EOAA, 150 μg mL^−1^ of 1,8‐cineole and 150 μg mL^−1^ of camphor. These treatments led to remarkable increases in ABA content, with fold‐changes of 20.32, 18.77 and 22.25, respectively, compared to the control [Fig. 4(b)]. The notable elevation of ABA, which is connected to dormancy and stress responses, provides additional evidence for the inhibitory effects of these compounds on seed germination.

### 3.5 Starch hydrolysis‐related parameters

**Figure 5:**
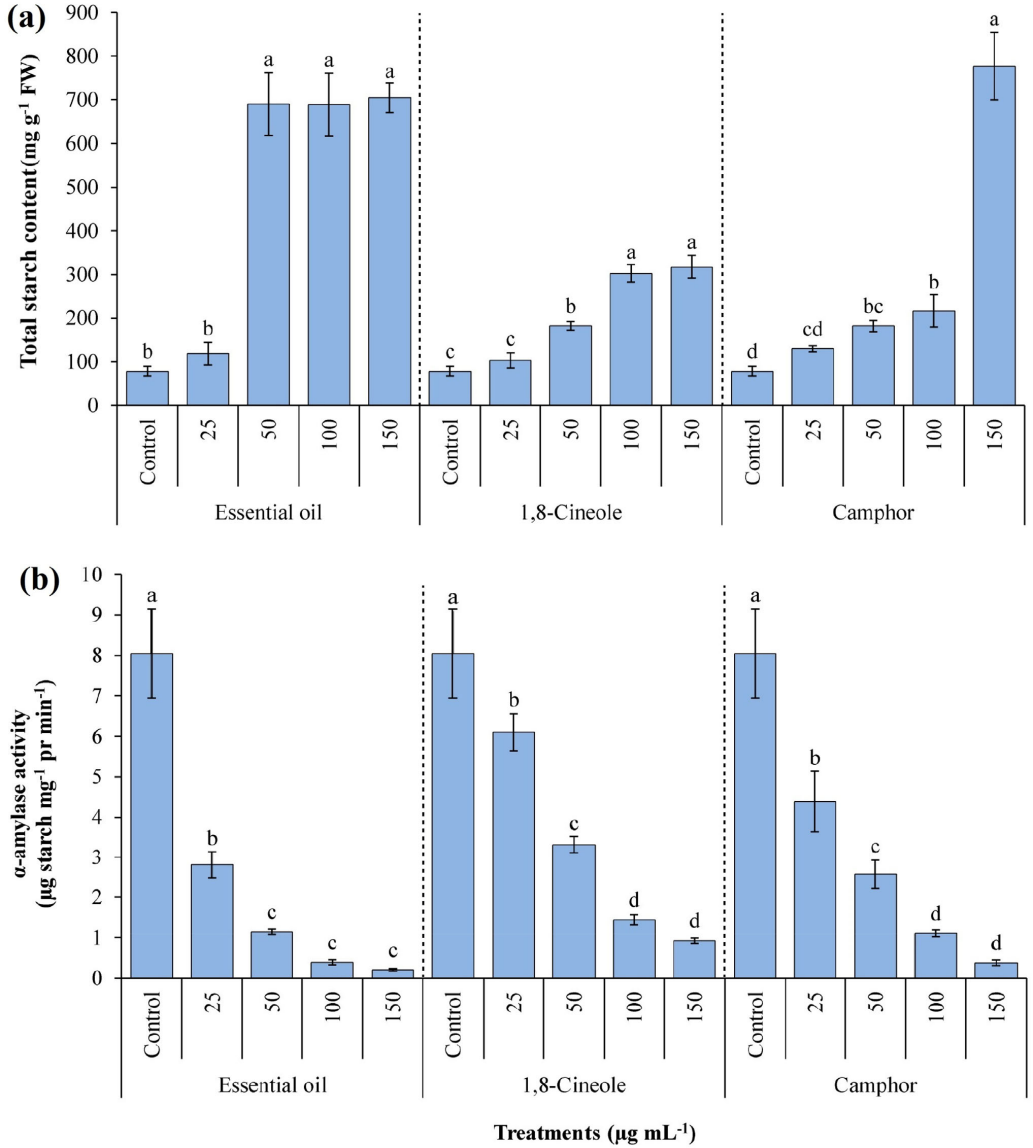
Content of total starch (a) and *α*‐amylase activity (b) in *Avena fatua* seeds when treated with various concentrations of essential oil from *Artemisia austriaca*, 1,8‐cineole and camphor. Different letters on each column indicate statistically significant differences at *P* ≤ 0.05 as determined by Duncan's multiple range test. The data are presented as the mean of three replicates ± standard deviation.

The analysis revealed that the total starch content in *A. fatua* seeds treated with various concentrations of EOAA, 1,8‐cineole and camphor was significantly higher (*P* ≤ 0.05) compared to the untreated control, particularly at concentrations of 50, 100 and 150 μg mL^−1^ (Fig. 5). The most evident accumulation of starch was observed at the highest concentration tested (150 μg mL^−1^) for all three treatments. Specifically, seeds treated with EOAA, 1,8‐cineole and camphor at this concentration exhibited increased starch content by 8.99‐, 4.05‐ and 9.92‐fold, respectively, relative to the control samples [Fig. 5(a)].

In parallel, the activity of *α*‐amylase, a key enzyme responsible for breaking down starch into simpler sugars, was significantly inhibited (*P* ≤ 0.05) by all applied concentrations of the volatile compounds [Fig. 5(b)]. At 150 μg mL^−1^ of EOAA, 1,8‐cineole and camphor, maximal suppression of *α*‐amylase activity was observed with reductions of 97.51%, 88.55% and 95.39%, respectively, compared to the control groups.

### 3.6 Total, reducing, and non‐reducing sugars

**Figure 6:**
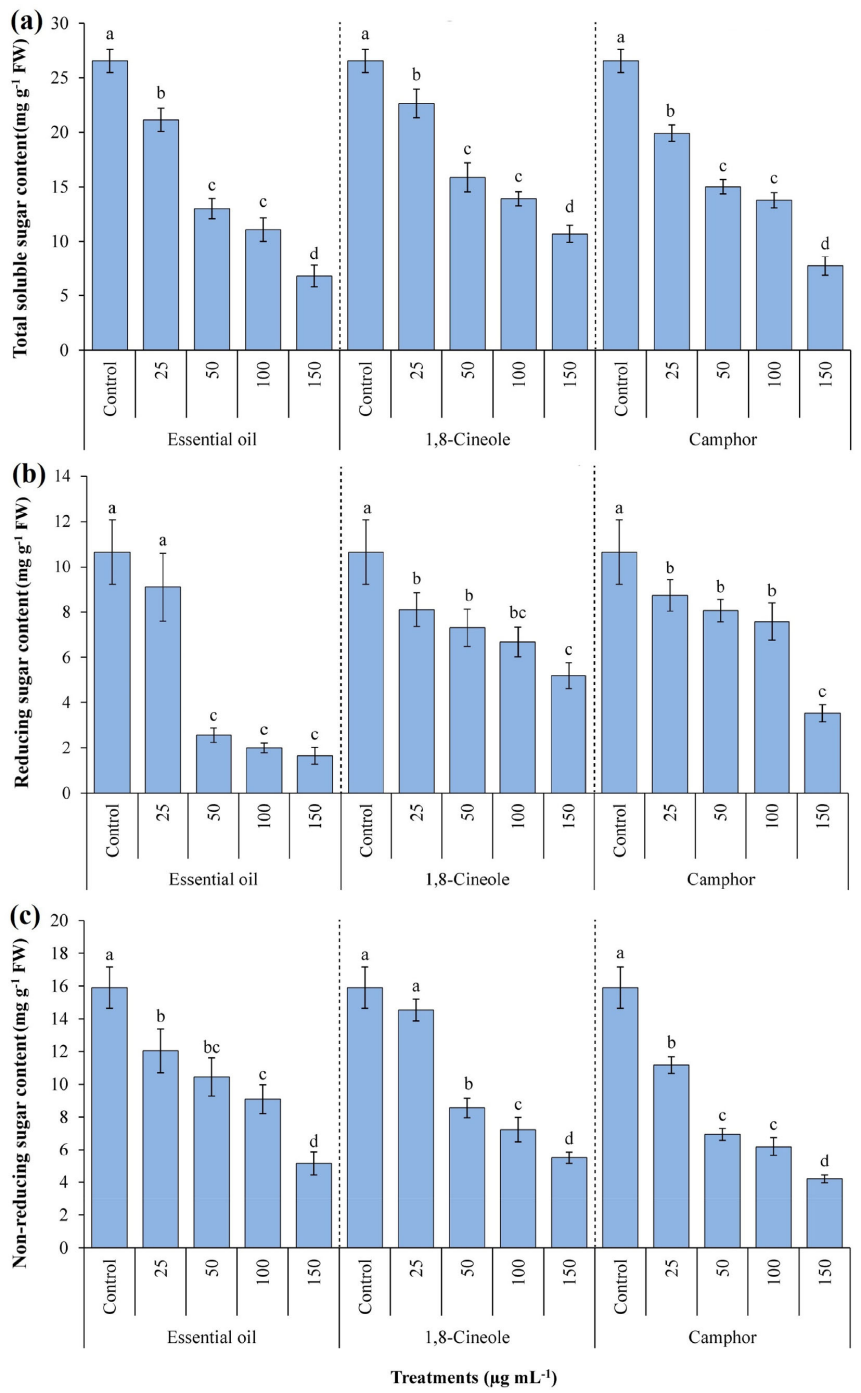
Content of total soluble sugars (a), reducing sugars (b) and non‐reducing sugars (c) in *Avena fatua* seeds when treated with various concentrations of essential oil from *Artemisia austriaca*, 1,8‐cineole and camphor. Different letters on each column indicate statistically significant differences at *P* ≤ 0.05 as determined by Duncan's multiple range test. The data are exhibited as the mean of three replicates ± standard deviation.

Figure 6 depicts the levels of total soluble sugars, as well as reducing and non‐reducing sugars in *A. fatua* seeds. All parameters exhibited a concentration‐dependent significant (*P* ≤ 0.05) decline with increasing concentrations of EOAA, 1,8‐cineole and camphor. The most significant reduction in total soluble sugars occurred at the concentration of 150 μg mL^−1^, showing reductions of 74.43%, 59.74% and 71.22% for each treatment, respectively, compared to the control samples. Likewise, the lowest levels of reducing sugars were observed at 150 μg mL^−1^ of EOAA, 1,8‐cineole and camphor, with decline factors of 84.56%, 51.22% and 66.91%, respectively. A maximum decrease in non‐reducing sugar content also was recorded at 150 μg mL^−1^, with reductions of 67.61%, 65.41% and 73.58%, respectively, compared to the control.

### 3.7 Lipid catabolism‐related traits

**Figure 7:**
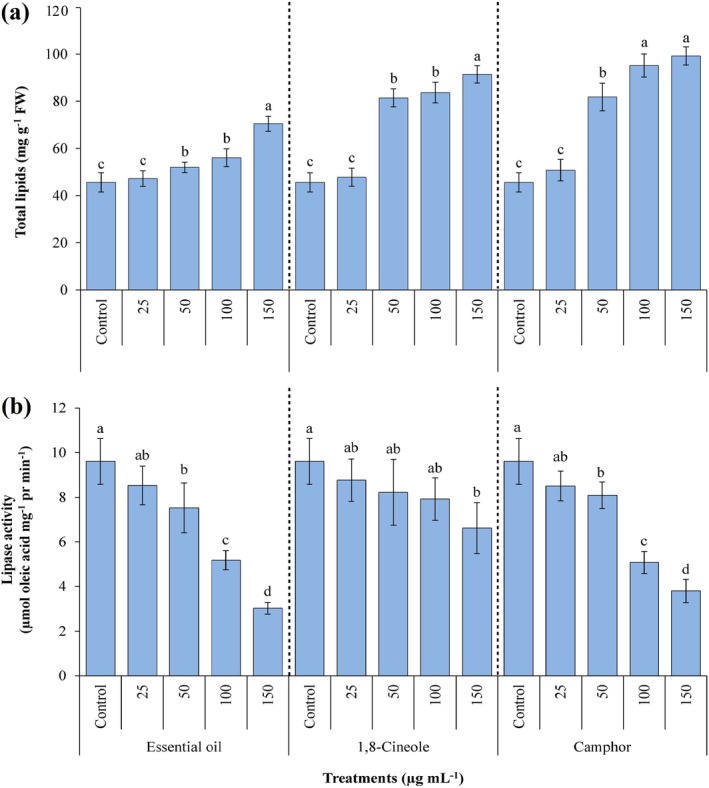
Content of total lipids (a) and lipase activity (b) in *Avena fatua* seeds when treated with various concentrations of essential oil from *Artemisia austriaca*, 1,8‐cineole and camphor. Different letters on each column indicate statistically significant differences at *P ≤* 0.05 as determined by Duncan's multiple range test. The data are displayed as the mean of three replicates ± standard deviation.

The results presented in Fig. 7 illustrate the effects of various concentrations of EOAA, 1,8‐cineole, and camphor on the total lipid content and lipase activity in *A. fatua* seeds. The data revealed significant changes in both parameters, highlighting the impact of these volatile compounds on lipid metabolism. The total lipid content was significantly higher (*P* ≤ 0.05) in samples treated with 50, 100 and 150 μg mL^−1^ of the volatile compounds compared to the untreated control. The most pronounced increases in lipid content were observed in seeds treated with 150 μg mL^−1^ of EOAA, 1,8‐cineole and camphor. These treatments resulted in increments of 54.44%, 2‐ and 2.17‐fold, respectively, relative to the control samples.

By contrast, lipase activity, which plays a crucial role in the breakdown of stored lipids into free fatty acids, was significantly reduced (*P ≤* 0.05) in seeds treated with the volatile compounds compared to the control samples. The lowest lipase activity was observed in seeds treated with 100 μg mL^−1^ of EOAA, 150 μg mL^−1^ of 1,8‐cineole and 150 μg mL^−1^ of camphor. These treatments led to reductions in lipase activity of 20.32%, 18.77% and 22.25%, respectively, compared to the control.

The fatty acid profile of *A. fatua* seeds exposed to various concentrations of EOAA, 1,8‐cineole and camphor revealed significant differences (*P* ≤ 0.05) in fatty acid quantity and quality compared to the control group (Table 4). The total fatty acids were significantly accumulated in a dose‐dependent manner when treated with 1,8‐cineole and camphor. The highest levels of fatty acids were recorded at a concentration of 150 μg mL^−1^ of 1,8‐cineole and camphor with increase factors of 86.69% and 87.62%, respectively, compared to the control.

**Table 4:** Fatty acid profile of *Avena fatua* seeds exposed to various concentrations of EOAA, 1,8‐cineole, and camphor

Treatments (μg mL^–1^)		Fatty acids (mg g^–1^ FW)					
		Palmitic acid	Linoleic acid	α‐Linolenic acid	Stearic acid	Oleic acid	Total
Essential oil	Control	26.68 ± 0.68a	3.33 ± 0.04d	4.39 ± 0.02a	2.12 ± 0.03b	5.48 ± 0.08c	42.00 ± 0.35a
	25	11.24 ± 0.17d	5.90 ± 0.07c	0.00 ± 0.00b	2.54 ± 0.04b	18.77 ± 0.27a	38.44 ± 0.45b
	50	21.35 ± 0.36b	9.28 ± 0.08b	0.00 ± 0.00b	4.15 ± 0.03a	4.18 ± 0.05c	38.96 ± 0.51b
	100	14.93 ± 0.24c	9.96 ± 0.07b	0.00 ± 0.00b	0.00 ± 0.00c	10.47 ± 0.06b	35.36 ± 0.38c
	150	16.24 ± 0.63c	12.92 ± 0.13a	0.00 ± 0.00b	0.00 ± 0.00c	10.59 ± 0.08b	39.75 ± 0.55b
1,8‐Cineole	Control	26.68 ± 0.68b	3.33 ± 0.04d	4.39 ± 0.02a	2.12 ± 0.03b	5.48 ± 0.08d	42.00 ± 0.35c
	25	20.00 ± 0.57c	11.75 ± 0.04c	0.00 ± 0.00b	0.00 ± 0.00c	5.94 ± 0.03d	37.69 ± 0.41d
	50	19.41 ± 0.25cd	35.12 ± 0.06a	0.00 ± 0.00b	6.58 ± 0.05a	13.86 ± 0.14c	74.97 ± 0.63b
	100	18.86 ± 0.07d	27.69 ± 0.04b	0.00 ± 0.00b	5.67 ± 0.06a	23.56 ± 0.33b	75.78 ± 0.67b
	150	37.72 ± 0.21a	0.00 ± 0.00e	0.00 ± 0.00b	0.00 ± 0.00c	40.69 ± 0.35a	78.41 ± 0.48a
Camphor	Control	26.68 ± 0.68a	3.33 ± 0.04c	4.39 ± 0.02a	2.12 ± 0.03c	5.48 ± 0.08d	42.00 ± 0.35c
	25	11.14 ± 0.10c	2.24 ± 0.01c	0.00 ± 0.00b	8.12 ± 0.07b	17.21 ± 0.05b	38.71 ± 0.46d
	50	23.43 ± 0.42b	24.63 ± 0.81b	0.00 ± 0.00b	0.00 ± 0.00d	30.60 ± 0.21a	78.66 ± 0.52a
	100	10.34 ± 0.08c	26.26 ± 0.72b	0.00 ± 0.00b	0.00 ± 0.00d	30.16 ± 0.33a	66.76 ± 0.47b
	150	5.62 ± 0.03d	38.53 ± 0.14a	0.00 ± 0.00b	24.09 ± 0.65a	10.57 ± 0.14c	78.80 ± 0.72a

Different letters in each column indicate statistically significant differences at *P* ≤ 0.05 as determined by Duncan's multiple range test. The data are presented as the mean of three replicates ± standard deviation.

### 3.8 Total soluble proteins

**Figure 8:**
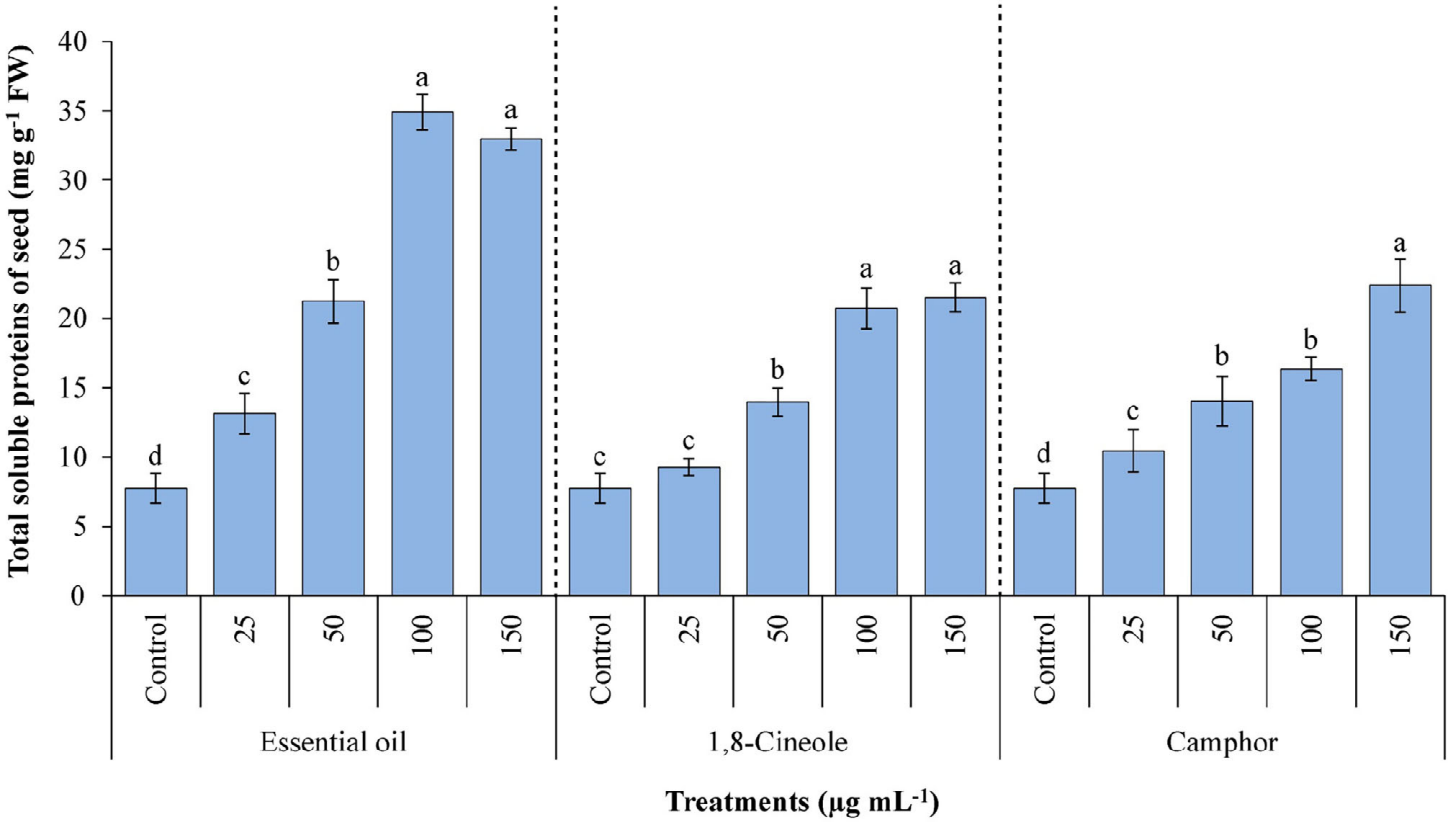
Content of total soluble proteins in *Avena fatua* when treated with various concentrations of essential oil from *Artemisia austriaca*, 1,8‐cineole and camphor. Different letters on each column indicate statistically significant differences at *P* ≤ 0.05 as determined by Duncan's multiple range test. The data are exhibited as the mean of three replicates ± standard deviation.

Figure 8 illustrates the total soluble protein content in *A. fatua* seeds exposed to various concentrations of EOAA, 1,8‐cineole and camphor. The total soluble protein content exhibited a concentration‐dependent increase as the levels of the volatile compounds rose. The maximum accumulation was achieved at 100 μg mL^−1^ of EOAA, 150 μg mL^−1^ of 1,8‐cineole and 150 μg mL^−1^ of camphor, with increases of 4.49‐, 2.77‐ and 2.88‐fold, respectively, compared to the control.

## 4 DISCUSSION

The chemical compositions of EOAA were investigated in the present study using HS‐SPME‐GC‐MS. The major compounds obtained by this method were comparable to those of our previous investigation, in which the chemical compositions of EOAA were analyzed via *n*‐hexane solvent extraction.^5^ Both methods identified *β*‐myrcene, 1,8‐cineole and camphor as the major constituents, demonstrating consistency in the qualitative profile. However, significant quantitative differences were observed. In the current HS‐SPME‐GC‐MS analysis, oxygenated monoterpenes such as camphor (26.52%) and 1,8‐cineole (20.15%) were detected at higher concentrations compared to the solvent extraction analysis (camphor: 21.62%; 1,8‐cineole: 13.45%). Likewise, sesquiterpenes like *δ*‐cadinene showed elevated levels in the present study (4.18%) relative to the solvent extraction method (2.47%). Previous investigations also demonstrated the SPME method's efficiency in detecting volatile agents as a consequence of its extraction competence, simplicity, low cost, and speed, implying higher recovery rates and higher sensitivity, as well as selectivity for volatile compounds.^27^, ^28^ For the higher recovery rate for the monoterpenes, the SPME technique was selected to detect possible volatiles absorbed by seeds treated with EOAA, 1,8‐cineole, and camphor.

The germination‐inhibitory properties of EOs have recently attracted significant interest owing to their potential application as bioherbicides. In our latest research, we found that the EOAA and its primary constituents, 1,8‐cineole and camphor, demonstrate substantial bioactivity against the challenging weed species *A. fatua*.^5^ Specifically, these compounds effectively suppress seed germination and inhibit seedling growth. The potential mechanisms underlying the inhibition of seedling growth include the induction of oxidative stress and a reduction in cell viability. However, the exact mechanisms responsible for germination suppression remain unclear and require further investigation. Although proposed mechanisms such as amylase inhibition by allelochemicals at the seed level exist, other pathways related to hormones, starch, storage lipids, and protein degradation are still not well‐understood.

**Figure 9:**
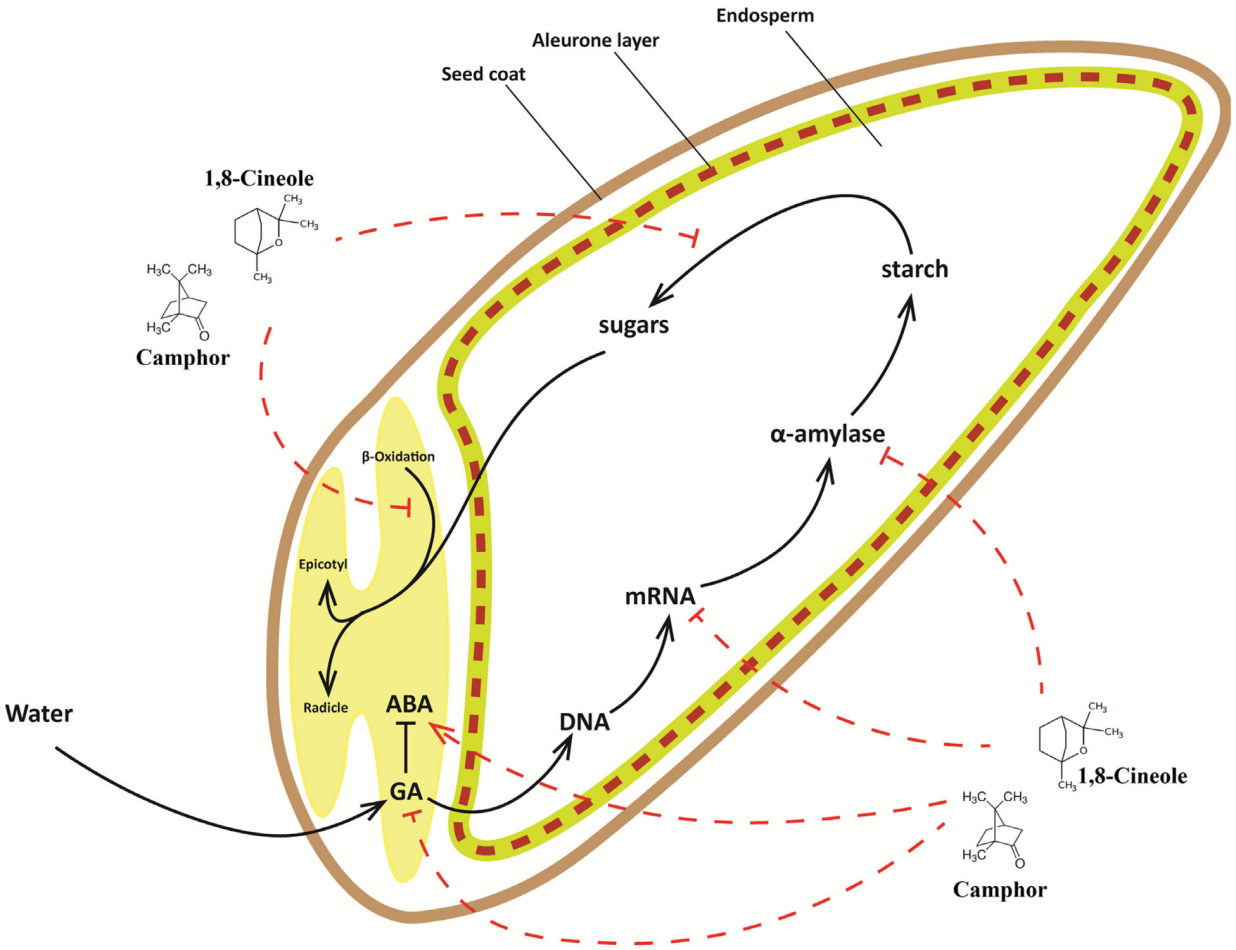
Schematic illustration of the germination pathway in *Avena fatua* seeds and possible mechanisms for its inhibition via terpenoid compounds.

Owing to their lipophilic nature, 1,8‐cineole and camphor can easily infiltrate cell membranes, which may lead to the disruption of seed germination in target plants by interfering with essential physiological and biochemical mechanisms. As depicted in Fig. 9, these compounds suppress *A. fatua* germination by penetrating seed tissues and modifying pathways critical for germination. Although the mechanisms of allelochemical absorption into seeds are still relatively unexplored, current evidence suggests that such penetration may indeed occur. For example, Dudai *et al*.^29^ demonstrated that terpenoids such as isomenthol and pulegone, which are volatile compounds released from the leaf debris of *Micromeria fruticosa* (L.) Druce, was found within exposed seeds. This finding validates their ability to pass through seed coats and accumulate inside. Likewise, Chiapusio *et al*.^30^ reported the uptake of the allelochemical 2‐benzoxazolinone (BOA) by radish seeds and seedlings, thereby emphasizing the idea that seeds are capable of absorbing bioactive substances from their environment. Collectively, these studies highlight the ability of terpenoids to function as strong germination inhibitors through the disruption of internal metabolic mechanisms.

During seed germination, the interaction between GA and ABA is crucial for regulating the transition from dormancy to active growth. ABA is a hormone that promotes dormancy, whereas GA activates the processes leading to the termination of dormancy and the onset of germination.^31^ Seeds carefully regulate the hormonal equilibrium between GA and ABA. The absorption of water by seeds leads to a reduction in ABA levels and an increase in GA levels, thereby triggering the germination process. The degradation of ABA holds particular significance owing to its role in inhibiting germination‐related genes, many of which are under the control of the transcription factor ABI5, an essential component of ABA signaling pathways.^32^, ^33^ The findings of our study indicate that monoterpenoids, when applied to *A. fatua* seeds, interfere with the hormonal equilibrium necessary for the germination process. The observed elevation in ABA levels, together with a significant reduction in GA contents, suggests that these compounds may have an inhibitory effect on seed germination. This disruption is likely to delay the activation of GA‐mediated pathways necessary for mobilizing stored reserves and initiating germination. Previous observations have indicated that allelochemicals can disrupt the balance of phytohormones and inhibit the process of germination. For instance, Li *et al*.^34^ demonstrated that maize (*Zea mays* L.) seeds subjected to faba bean (*Vicia faba* L.) extracts, which are rich in allelochemicals, exhibited increased levels of ABA and decreased levels of GA when compared to untreated control seeds. This observation supports the hypothesized mechanism of the action of 1,8‐cineole and camphor in *A. fatua*. These findings emphasize the potential of allelochemicals, such as monoterpenoids, to target hormonal pathways for suppressing germination.

The cereal seeds contain a starchy endosperm, which is essential for providing carbohydrates to germinating seeds. This endosperm is covered by an aleurone layer, where *α*‐amylase is synthesized and secreted.^35^ The induction mediated by GA, initiates the production of hydrolyzing enzymes, particularly α‐amylase, which catalyzes the hydrolysis of starch into simpler sugars. These sugars serve as vital energy sources that drive the growth of embryos and facilitate the initial development of seedlings.^15^ It has been proposed that the inhibition of germination caused by allelochemicals may be linked to a decrease in *α*‐amylase activity.^36^ *In vitro* studies also have confirmed that terpenoid‐rich EOs, such as those containing camphor and 1,8‐cineole isolated from *Artemisia herba‐alba* ^37^ and *Curcuma caesia* Roxb.,^38^ have an inhibitory effect on *α*‐amylase activity. Monoterpenoids may bind to critical catalytic residues in the enzyme's active site, such as Arg195 and Asp197, blocking substrate access and impairing starch hydrolysis.^39^

The inhibition of *α*‐amylase activity significantly affects the germination of *A. fatua* seeds. The decreased breakdown of starch results in its accumulation within the seed, as seen in this study, where starch content increased notably. At the same time, the levels of reducing sugars remarkably declined. The decrease in soluble sugars corresponds with the reduced activity of amylolytic enzymes, including *α*‐amylase, *β*‐amylase, and glycosidases. These enzymes are essential for breaking down starch after seed imbibition. The resulting lack of energy prevents the embryo from accessing the metabolic resources necessary for germination consequently delaying or ceasing the process.^40^, ^41^

Seeds are known as reservoirs for proteins and polysaccharides, which are utilized during germination to release the essential compounds needed for the growth and development of young plants.^42^ In this study, exposing *A. fatua* seeds to volatile monoterpenoids resulted in an accumulation of total soluble proteins, indicating that proteolysis was hindered. Proteases generally catalyze the hydrolysis of seed storage proteins into polypeptides, which are then further degraded into amino acids by peptidases. This process guarantees a sufficient availability of components necessary for the synthesis of new proteins.^15^, ^43^ It has been suggested that GA stimulates proteolytic enzymes for the degradation of seed storage proteins.^44^ Monoterpenoids can inhibit GA biosynthesis and reduce the secretion of proteolytic enzymes in the endosperm, leading to an increased persistence of proteins in seeds. Additionally, these compounds may act as inhibitors of proteases and peptidases, thereby preventing the consumption of proteins.

In addition to storing starch and storage proteins, seeds also accumulate triacylglycerols (TAG), which function as storage lipids and serve as an energy source for the growth of seedlings during germination.^45^ When seeds absorb water, TAGs are broken down by lipases into fatty acids and glycerol. The fatty acids are then transported to peroxisomes, where they undergo the *β*‐oxidation process to be converted into acetyl‐CoA.^46^, ^47^ Acetyl‐CoA is converted into sugars through a range of metabolic pathways, including the glyoxylate cycle, the tricarboxylic acid cycle and gluconeogenesis. The initial step in the breakdown of TAG involves the hydrolysis process catalyzed by lipases. This process is crucial for providing a carbon source to the embryo, particularly because photosynthesis has not yet begun.^48^ The results obtained demonstrate that exposure of *A. fatua* seeds to monoterpenoids reduces their lipase activity. This may be related to the direct inhibitory effects of 1,8‐cineole and camphor on lipase enzymes. There is currently no information on the effects of allelochemicals such as terpenoids, phenolics, and alkaloids on lipase activity in phytotoxicity bioassays. However, there is some evidence indicating that EOs derived from certain plant species may inhibit lipase activity.^49^, ^50^ It has been proposed that the compounds contained in EOs may inhibit lipase activity through a non‐competitive mechanism, although the specific details of this mechanism remain unknown.^51^ The inhibition of lipase activity and the subsequent accumulation of fatty acids in *A. fatua* seeds exposed to EOAA, 1,8‐cineole, and camphor highlight the disruptive effects of these volatile compounds on fatty acid and lipid metabolism. The accumulation of total fatty acids in the seeds suggests that the *β*‐oxidation process may be hindered by volatile compounds. However, a deeper study on *β*‐oxidation and related procedures is necessary for understanding the fundamental mechanisms involved.

Although our laboratory data showed potent inhibition of *A. fatua* germination, future work must test whether these monoterpenoids at field‐applicable concentrations affect germination of major cereal crops (e.g. *Triticum aestivum* and *Hordeum vulgare*). The lipophilic nature of camphor and 1,8‐cineole also suggests possible effects on non‐target seeds or soil microbes; thus, dosage optimization and encapsulation formulations should be considered. Furthermore, given the high volatility of camphor and 1,8‐cineole, direct applications would be likely to dissipate within hours. Thus, encapsulation is suggested for more stability of the volatile compounds in future applications especially in field practice.

## 5 CONCLUSIONS

The findings of this study provide comprehensive insights into the multifaceted mechanisms through which EOAA, 1,8‐cineole, and camphor inhibit the germination of *A. fatua* seeds. These volatile compounds are adsorbed by seed tissues, as confirmed by HS‐SPME‐GC‐MS analysis, and disrupt fundamental physiological and biochemical processes for the germination. The observed suppression of GA and elevation of ABA levels indicate these compounds interfere with hormonal signaling pathways, tipping the balance toward dormancy. Additionally, the inhibition of key enzymes such as *α*‐amylase and lipase disrupts the mobilization of starch and lipid reserves, leading to the accumulation of starch, fatty acids and total lipids. This metabolic disruption deprives the embryo of necessary energy and essential building blocks, effectively halting its growth and development. Furthermore, the selective modulation of fatty acid profiles confirms interference with lipid biosynthesis and *β*‐oxidation pathways. These results align with the broader allelopathic actions of bioactive compounds, which utilize hormonal and metabolic deficiencies to suppress seedling establishment. Collectively, these findings underscore the potential of camphor and 1,8‐cineole as natural herbicides or allelopathic agents for weed management. However, further research is needed to elucidate their precise molecular interactions, uptake mechanisms, and downstream effects on cellular metabolism. Such knowledge will be essential for developing targeted and environmentally sustainable strategies to control weeds such as *A. fatua*, to minimize ecological impacts.

## CONFLICT OF INTEREST

The authors declare that they have no known competing financial interests or personal relationships that could have appeared to influence the work reported in this paper.

## Data Availability

The data that support the findings of this study are available from the corresponding author upon reasonable request.
